# Oxidation of Dichloromethane over Au, Pt, and Pt-Au Containing Catalysts Supported on γ-Al_2_O_3_ and CeO_2_-Al_2_O_3_

**DOI:** 10.3390/molecules25204644

**Published:** 2020-10-12

**Authors:** Tuomas K. Nevanperä, Satu Pitkäaho, Satu Ojala, Riitta L. Keiski

**Affiliations:** Faculty of Technology, Environmental and Chemical Engineering, University of Oulu, P.O. Box 4300, FI-90014 Oulu, Finland; tuomas.nevanpera@oulu.fi (T.K.N.); satu.pitkaaho@oulu.fi (S.P.); satu.ojala@oulu.fi (S.O.)

**Keywords:** environmental catalysis, gold, platinum, bimetallic catalyst, chlorinated volatile organic compound (CVOC)

## Abstract

Au, Pt, and Pt-Au catalysts supported on Al_2_O_3_ and CeO_2_-Al_2_O_3_ were studied in the oxidation of dichloromethane (DCM, CH_2_Cl_2_). High DCM oxidation activities and HCl selectivities were seen with all the catalysts. With the addition of Au, remarkably lower light-off temperatures were observed as they were reduced by 70 and 85 degrees with the Al_2_O_3_-supported and by 35 and 40 degrees with the CeO_2_-Al_2_O_3_-supported catalysts. Excellent HCl selectivities close to 100% were achieved with the Au/Al_2_O_3_ and Pt-Au/Al_2_O_3_ catalysts. The addition of ceria on alumina decreased the total acidity of these catalysts, resulting in lower performance. The 100-h stability test showed that the Pt-Au/Al_2_O_3_ catalyst was active and durable, but the selectivity towards the total oxidation products needs improvement. The results suggest that, with the Au-containing Al_2_O_3_-supported catalysts, DCM decomposition mainly occurs via direct DCM hydrolysis into formaldehyde and HCl followed by the oxidation of formaldehyde into CO and CO_2_.

## 1. Introduction

Chlorinated volatile organic compounds (CVOCs) are widely used in the industry as solvents, degreasing and cleaning agents, and paint strippers and as additives for paints, inks, and adhesives, as well as raw materials in the synthesis of chemicals, plastics, and pharmaceuticals [1,2,3]. Dichloromethane (CH_2_Cl_2_, DCM) was chosen as the model CVOC in this work. DCM is one of the three main chlorinated solvents used in Europe, the others being perchloroethylene (PCE) and trichloroethylene (TCE) [4]. DCM is the most stable chlorinated alkane, and therefore, it is very difficult to be decomposed naturally in the environment [5]. The 100-year global warming potential (GWP) value for DCM is nine [6]. In the International Chemical Safety Cards (ICSC), the effects of DCM are identified as: irritating to the eye, skin, and respiratory tract; causing effects on the central nervous system, blood, liver, heart, and lungs; exposure at high concentrations causing a lowering of consciousness and death; and, probably, being a carcinogenic to humans [7].

Among the end-off pipe technologies, catalytic oxidation is a cost-effective and environmentally friendly destructive method for the abatement of VOCs [2,8,9]. In CVOC oxidation, selectivity of the catalyst is of great importance to ensure the production of HCl while restraining the formation of byproducts and, also, minimizing the deactivation of the catalyst. Besides choosing a durable and selective catalyst, ensuring an adequate hydrogen amount in the reaction is important [10,11,12,13,14,15,16,17,18,19,20]. In our previous study [12], we used water as a hydrogen source, and its amount was optimized to be 1.5 vol.%. Among the 15 metallic monoliths containing Pt, Pd, Rh, and V_2_O_5_ supported on alumina, alumina-titania, and alumina-ceria, 1 wt.% Pt/Al_2_O_3_ proved to be the most active and HCl selective catalyst, showing 100% DCM conversion at 420 °C and over 90% HCl yield at above 540 °C. In addition, the Al_2_O_3_-CeO_2_-supported catalysts showed good activity in DCM oxidation and enhanced selectivity towards CO_2_, but the HCl yields remained below 90% at the temperature range used in the experiments. [12] In general, platinum group metals have proven to be highly active and durable catalysts for the oxidation of CVOCs [2,9,21].

In order to develop even more efficient, selective, and sustainable catalysts, the suitability of gold catalysts in CVOC oxidation also needs to be investigated. Aida et al. [22] studied Au catalysts having different loadings on a number of metal oxides in methyl chloride (CH_3_Cl) oxidation. Another study by Aida et al. [23] included experiments with 1.4 wt.% Au/TiO_2_ catalyst in methyl chloride oxidation and 5 wt.% Au/Al_2_O_3_ catalyst in methyl chloride, DCM, chloroform (CHCl_3_), and carbon tetrachloride (CCl_4_) oxidation. Based on these studies [22,23], Aida et al. concluded that, after some stability improvement, the Au/Al_2_O_3_ catalyst could be a promising catalyst for the decomposition of halogenated organic compounds due to its high activity and HCl selectivity. Chen et al. [24] investigated DCM oxidation using Au/Co_3_O_4_, Co_3_O_4_, Cr_2_O_3_/Al_2_O_3_, Pd/Al_2_O_3_, and Pt/Al_2_O_3_ catalysts. Among all the tested catalysts, 0.2 wt.% Au/Co_3_O_4_ was the best, showing complete DCM conversion at 350 °C. In addition, after a 130-h stability test, the catalyst proved its durability in laboratory conditions. [24] Matějová et al. [25] studied Au and Pt catalysts supported on ceria-zirconia in the total oxidation of DCM. They concluded that, even though the support itself showed high activity, the noble metal catalysts exhibited significantly enhanced selectivity towards CO_2_ compared to the ceria-zirconia support alone, gold catalysts showing somewhat lower selectivity than platinum in this case. [25] Redina et al. [26] studied the performance of an Au-Rh/TiO_2_ catalyst in DCM oxidation, which was prepared using surface redox reactions. In this study, bimetallic catalysts having metal contents as low as 0.05 wt.% showed high activity and HCl selectivity. In addition, the selectivity towards CO_2_ formation was enhanced when compared to a monometallic Au catalyst with the same Au content. However, strong conclusions on the long-term stability of the catalyst cannot be drawn, because the authors reported an onstream time of only 190 min [26].

In this study, the catalytic performance and selectivity of monometallic Au and bimetallic Pt-Au catalysts supported on alumina and ceria-alumina were studied in dichloromethane (DCM, CH_2_Cl_2_) oxidation. In addition, the durability of the Pt-Au/Al_2_O_3_ catalyst was studied by performing a 100-h stability test. Monometallic Pt catalysts, i.e., Pt/Al_2_O_3_ and Pt/CeO_2_-Al_2_O_3_, were used as references, as they have proven to be active and selective catalysts in DCM oxidation. To explain the performance of the catalysts, they were characterized by inductively coupled plasma optical emission spectroscopy (ICP-OES), N_2_ physisorption, X-ray diffraction (XRD), X-ray photoelectron spectroscopy (XPS), high-resolution transmission electron microscope (HR-TEM), temperature-programmed reduction with hydrogen (H_2_-TPR), temperature-programmed desorption of ammonia (NH_3_-TPD), and carbon dioxide (CO_2_-TPD), and temperature-programmed ^18^O_2_ isotopic exchange (^18^O_2_-TPIE). Based on the results, suggestions related to the reaction mechanism are made for an Au/Al_2_O_3_ catalyst.

## 2. Results and Discussion

Table 1 shows the results previously published in References [27,28] and the new results presented in this study. More detailed characterization results are presented and discussed in References [27,28], i.e., for monometal and support ICP-OES, BET (Brunauer-Emmett-Teller method), XRD, H_2_-TPR, and ^18^O_2_-TPIE results in Reference [27] and for bimetal ICP-OES, BET-BJH (Brunauer-Emmett-Teller/Barrett-Joyner-Halenda method), XRD, XPS, HR-TEM, H_2_-TPR, NH_3_-TPD, CO_2_-TPD, and ^18^O_2_-TPIE results in Reference [28].

### 2.1. Properties of the Catalysts

Metal loadings, BET surface areas (S_BET_), pore volumes and average pore sizes, reducibility, and basicity of the catalysts are summarized in Table 2. Considering the CeO_2_-Al_2_O_3_ support, the desired weight ratio of 1:4 of ceria to alumina was verified by ICP-OES and XRF. ICP-OES showed 20.6 wt.% of ceria and 79.4 wt.% of alumina, whereas XRF demonstrated 21.7 wt.% and 78.3 wt.%, respectively [27].

**Figure 1:**
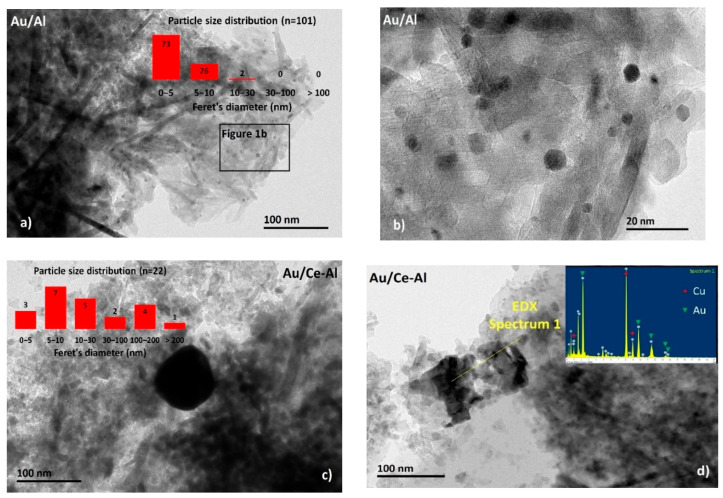
High-resolution transmission electron microscope (HR-TEM) images of the Au/Al_2_O_3_ and Au/CeO_2_-Al_2_O_3_ catalysts: (**a**) corresponding particle size distribution (PSD) data of Au/Al_2_O_3_, (**b**) example particles in sizes below 10 nm shown with the magnification of the inset in Figure 1a, (**c**) corresponding PSD data of Au/CeO_2_-Al_2_O_3_, and (**d**) an energy-dispersive X-ray (EDX) spectrum from the agglomerate (Cu peaks in the spectrum originate from the used sample grid).

**Figure 2:**
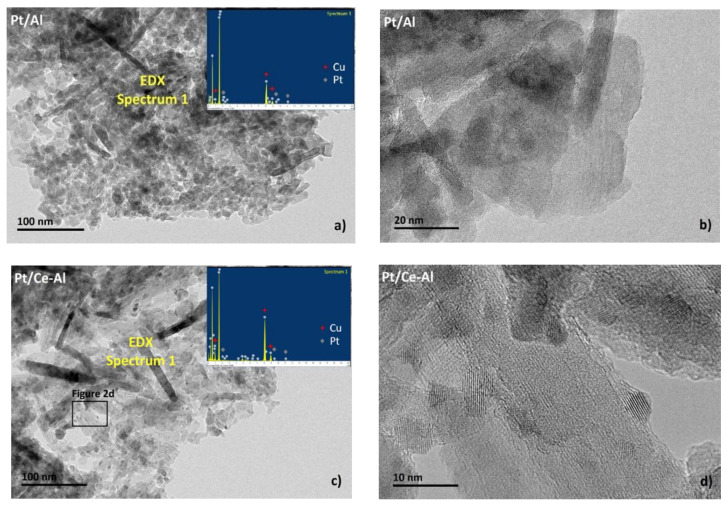
HR-TEM images of the Pt/Al_2_O_3_ and Pt/CeO_2_-Al_2_O_3_ catalysts: (**a**) EDX spectrum from the surface of Pt/Al_2_O_3_, (**b**) an example of a 20-nm scale image from the surface of Pt/Al_2_O_3_, (**c**) an EDX spectrum from the Pt/CeO_2_-Al_2_O_3_ surface, and (**d**) an example of a 10-nm scale image showing the magnification from the inset in Figure 2c. (Cu peaks in the EDX spectrum originate from the used sample grid.).

In Figure 1 and Figure 2, representative HR-TEM images of the prepared monometallic Au/Al and Au/Ce-Al catalysts with particle size distribution (PSD) and examples of energy-dispersive X-ray (EDX) spectra are presented. The HR-TEM results of bimetallic Pt-Au/Al and Pt-Au/Ce-Al can be found in Reference [28] and the Supplementary Materials. The PSD in each catalyst was calculated based on Feret’s diameter because of the irregular shape of the particles. Structures resembling needles in the HR-TEM images are side views of alumina 2D plates.

The Au/Al catalyst demonstrated homogeneously dispersed Au particles mainly in sizes smaller than 10 nm (Figure 1a,b). The majority of measured particles were below 5 nm. An EDX spectrum from the surface of Au/Al catalyst evidenced 1.8 wt.% of Au. The Au particles in the Au/Ce-Al catalyst were much larger when compared to the Au/Al catalyst (Figure 1c,d), as already discussed in Reference [27]. These findings were supported by the XRD and H_2_-TPR results. In comparison with the Au/Al catalyst, the PSD data of the Au/Ce-Al catalyst in Figure 1c show a low number of distinguishable particles (*n* = 22) that could be accurately measured and, therefore, do not represent a satisfactory population for PSD. The Au/Ce-Al catalyst contained unevenly dispersed particles in smaller sizes similarly to the Au/Al catalyst but, also, large agglomerates of fused particles in sizes of hundreds of nanometers likely due to sintering (Figure 1d). Islands of smaller particles that were close to fusing together were observed as well, which supports the occurrence of sintering. These particles were difficult to distinguish from each other and, therefore, could not be included in the PSD data. The EDX spectrum from the agglomerate in Figure 1c demonstrates intense Au peaks. Due to the very poor Au distribution, a representative bulk loading using EDX was not possible.

Figure 2a–d presents typical HR-TEM images of the Pt/Al and Pt/Ce-Al catalysts. Pt particles were not distinguished clearly from the surface due to contrast similarity, but the EDX spectra (Figure 2a,c) show evidence of Pt. The Pt particle size is below 10 nm based on Figure 2a–d and other interpreted images.

The Pt-Au/Al catalyst contained quite evenly dispersed particles, mostly in sizes between 10 to 30 nm (88%, *n* = 253), but some larger agglomerates were also observed. The Pt-Au/Ce-Al catalyst contained homogenously dispersed particles mostly in sizes between 10 to 30 nm (77%, *n* = 112) but, also, in sizes of 30–40 nm (11%). In addition, some larger isolated islands of agglomerated particles were observed. See Reference [28] for detailed information.

**Figure 3:**
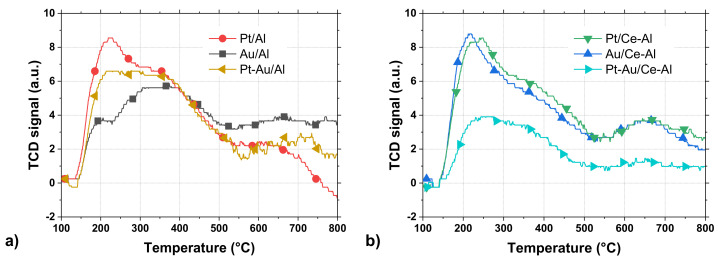
Temperature-programmed desorption of ammonia (NH_3_-TPD) profiles of the Pt, Au, and Pt-Au catalysts supported on (**a**) Al_2_O_3_ and on (**b**) CeO_2_-Al_2_O_3_, (TCD = thermal conductivity detector).

The total acidity of the catalysts was analyzed by NH_3_-TPD experiments. The temperature range of up to 600 °C is especially interesting, since it corresponds to the maximum temperature used in the light-off experiments and in the calcination step of catalyst preparation. The obtained NH_3_-TPD profiles are shown in Figure 3. In this study, the strengths of the acid sites are determined as weak and medium/strong in the sites retaining NH_3_ at lower than 300 °C and at higher than 300 °C, respectively. The shapes of the profiles and positions of the peaks indicate the presence of acid sites of different strengths, but one main band for all the catalysts centered at 210–250 °C corresponding to the weak acid sites can be seen in Figure 3. The Pt/Al, Au/Ce-Al, and Pt/Ce-Al catalysts show the highest amounts of weak acid sites, while the Pt-Au/Al, Au/Al, and Pt-Au/Ce-Al catalysts possess the lowest amounts. The amounts of strong acid sites are rather comparable between the catalysts, except in the case of Pt-Au/Ce-Al (see Figure 3b), which can, overall, be considered as the least acidic catalyst.

The basicity of the catalysts was measured to find out if it could explain the results related to CO_2_ formation during DCM oxidation. The total basicity of the catalysts was analyzed by CO_2_-TPD experiments; all results and, also, the new ones for monometallic catalysts, are summarized in Table 2. The CO_2_ uptake of the Al_2_O_3_-supported catalysts was between 100 to 135 μmol g^−1^ and the CeO_2_-Al_2_O_3_-supported catalysts between 85 to 160 μmol g^−1^. Au containing catalysts had higher total basicity than the corresponding Pt catalysts. The basicity of the catalysts was at a low level based on the CO_2_-TPD [29], which should be advantageous for the desorption of formed CO_2_ from the catalyst surface during DCM oxidation. The basic sites can be assigned low, medium, and high according to different CO_2_ desorption ranges at 80–140 °C, 160–240 °C, and >300 °C, respectively [30]. Based on our results, the Pt and Pt-Au catalysts lacked medium-strength basic sites, whereas the Au/Al and Au/Ce-Al catalysts had low, medium, and high-strength basic sites.

The reducibility of the catalysts was analyzed by H_2_-TPR, and the total hydrogen uptakes between 35 °C and 400 °C from References [27,28] are summarized in Table 2. Based on the H_2_ consumption, the Pt-Au/Al catalyst showed the lowest and Pt/Ce-Al the highest reducibility. Au/Al and Au/Ce-Al did not show significant reduction in the used temperature range, and the H_2_ consumption remained at a level of less than 50 μmol g^−1^. The Pt-Au/Al and Pt/Al catalysts demonstrated both H_2_ uptakes at a low temperature window of 50–70 °C and within a broad temperature range of 150–300 °C, both being caused likely by the reduction of adsorbed oxygen species and/or platinum oxychloride complexes. The TPR profiles of Pt-Au/Ce-Al and Pt/Ce-Al catalysts showed the H_2_ uptake maxima at 255 °C and at 205 °C, which can be assigned to the reduction of Pt species and surface ceria due to a hydrogen spillover enhanced by Pt particles [31]. The addition of Au in Pt catalysts decreased the reducibility, while the addition of ceria in the support improved the reducibility. Au might block the H_2_ spillover on Pt due to the preparation method used.

**Figure 4:**
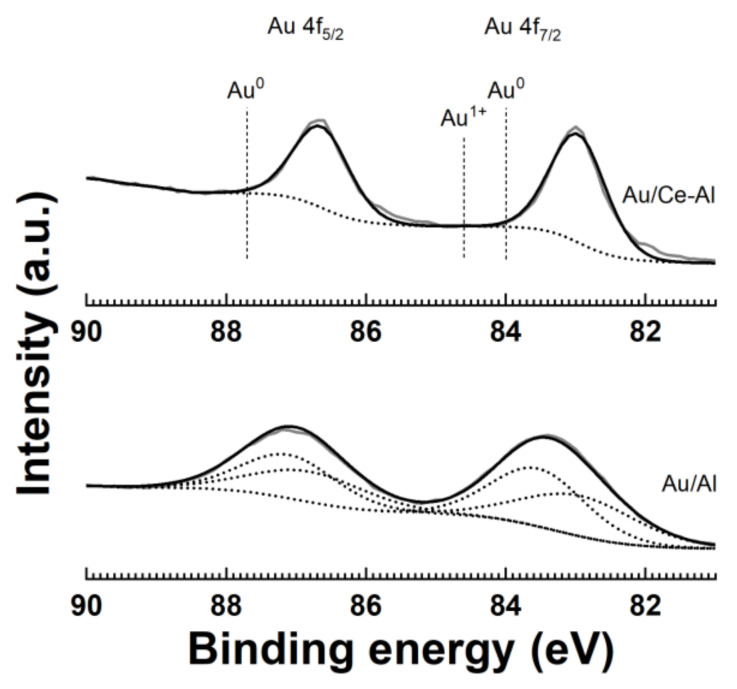
Au 4f spectra of the Au/Al and Au/Ce-Al catalysts (Au^0^ is the theoretical binding energy value of Au^0^).

XPS measurements were done to identify the oxidation state of Au in the Au/Al and Au/Ce-Al catalysts. A typical accuracy of the XPS measurements was 0.1 to 0.2 eV. The XPS results for the bimetallic catalysts (Pt-Au/Al and Pt-Au/Ce-Al) were presented in Reference [28]. A theoretical intensity ratio of I(Au 4f_7/2_):I(Au 4f_5/2_) = 4:3 for Au^0^ was used as one of the constraints in the fitting of the data [32]. Figure 4 shows the binding energy of the Au 4f spectra for the Au/Al and Au/Ce-Al catalysts. Usually, the oxidation states of Au are determined from the Au 4f_7/2_ at roughly 83.9–84.0 eV [33,34,35]. The characteristic peaks for different Au oxidation states are as follows: Au^0^ at 84.0 eV, Au^1+^ at 84.6 eV, and Au^3+^ at 85.9 eV [36,37]. Metallic Au^0^ is evidenced here based on the main peaks of Au 4f_7/2_ at 83.0 and 83.4 eV and Au 4f_5/2_ at 86.6 and 87.2. The binding energy values were lower than those of the bulk metallic Au, i.e., Au 4f_7/2_ = 84.0 eV and Au 4f_5/2_ = 87.7 eV. Similar results were reported earlier for Au^0^ on the surfaces of different catalysts. [37,38,39,40] These differences can be caused by the size-dependent peak shifts, the presence of hydroxides or oxides [37], and the metal-support and/or metal-metal interactions [37,38,39]. In addition, the Au/Al catalyst evidenced the presence of Au^1+^ based on the peak at 83.6 eV, which is supported by the known peak difference of 0.6 eV between the Au^0^ and Au^+1^ oxidation states [36,37]. In our case, the differences in peak positions of Au between mono- and bimetallic catalyst counterparts were relatively small. The peak shapes were similar, and the binding energy did not differ much. Au was in the metallic state in all Au-containing catalysts, except in the Au/Al catalyst that contained also Au^1+^.

Table 3 shows the surface composition and surface metal loading of the catalysts. Pt and Au contents close to the surface were higher compared to the bulk metal loadings analyzed by ICP-OES, which could be explained by the very low Au loading and the accuracy of the measurements. In the case of the Au/Al catalyst, the surface Au loading was 1.6 wt.% based on XPS, which is in accordance with the EDX result evidenced in HR-TEM (1.8 wt.%). Considering the CeO_2_-Al_2_O_3_ support, the bulk Al/Ce weight ratio was about 2.5 in comparison to the surface Al/Ce weight ratio of 7.4 and 10.9 for the Au/Ce-Al and Pt-Au/Ce-Al catalysts, respectively. Since the concentration of ceria on the surface is higher than in the bulk Au, it can interact with ceria more easily [41].

### 2.2. Catalytic Performance in DCM Oxidation

**Figure 5:**
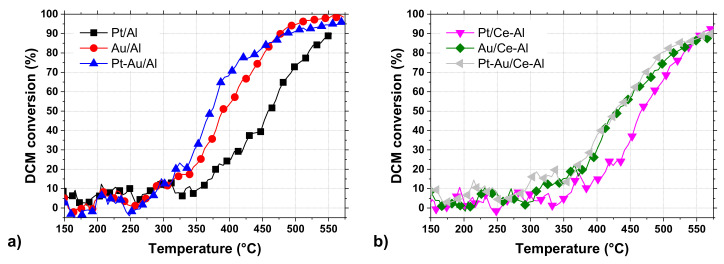
Dichloromethane (DCM) conversion of the Pt, Au, and Pt-Au catalysts supported on (**a**) Al_2_O_3_ and on (**b**) CeO_2_-Al_2_O_3_ (DCM 500 ppm, H_2_O 1.5 vol.%, weight hourly space velocity (WHSV) 720 g g_cat_^−1^ h^−1^).

The conversion curves of all the studied catalysts in DCM oxidation are presented in Figure 5a,b. The T_50_ (temperature at which 50% DCM conversion is observed) and T_90_ (temperature at which 90% DCM conversion is observed) values, together with the HCl and CO_2_ yields at T_90_ and at the maximum, are listed in Table 4. The results show that the bimetallic Pt-Au/Al and monometallic Au/Al catalysts were the most active catalysts among the tested catalysts (Figure 5a) based on their T_50_ and T_90_. The addition of Au resulted in a noticeable improvement in the performance by lowering the T_50_ by 65 °C and 85 °C in comparison to the γ-Al_2_O_3_ support and Pt/Al catalyst, respectively. When considering the catalysts supported on CeO_2_-Al_2_O_3_ (Figure 5b), similar results can be seen, i.e., gold in the catalyst improving the performance in DCM oxidation. The T_50_ was lowered by 35 °C and 40 °C in comparison to the CeO_2_-Al_2_O_3_ support and Pt/Ce-Al catalyst, respectively. It is noticeable that the active metal loadings in the Au/Al and Au/Ce-Al catalysts were 0.7 wt.% and 0.5 wt.%, respectively, which are much lower when compared to the Pt/Al catalyst with 1.1 wt.% and Pt/Ce-Al with 1.2 wt.% (see Table 2). In addition, the BET surface areas were quite comparable, being from 95 to 105 m^2^ g^−1^ for the Al_2_O_3_-supported catalysts and from 75 to 85 m^2^ g^−1^ for the CeO_2_-Al_2_O_3_-supported catalysts. Therefore, the better performance of Au-containing catalysts cannot be explained by the differences in these physical properties.

In this study, the presence of Au, alone or together with Pt, enhanced the performance of the catalyst compared to the catalyst containing only Pt. The addition of Pt improved the selectivity towards the total oxidation products but not the conversion, which is in agreement with the previous literature.

According to Maupin et al. [19], Pt/Al_2_O_3_ catalysts oxidize DCM completely at 380 °C, and the rate-limiting step takes place on alumina, since neither the loading of Pt nor the dispersion (particle size) have an effect on the conversion rates and selectivity. Similar observations were seen in our previous study [12]. The addition of Pt and/or ceria to the catalyst enhanced the selectivity towards CO_2_, but DCM conversion was not influenced significantly [12].

The activity of Au in oxidation reactions is known to be dependent on the particle size and shape [42,43]. The activation of oxygen by Pt occurs faster on larger particles [44]. The Au/Al catalyst contained well-dispersed nanoparticles in sizes below 10 nm, whereas, in the Pt-Au/Al catalyst, the particles were mainly in sizes between 10 to 30 nm. In addition, the Pt-Au/Ce-Al catalyst had mostly particles in sizes between 10 to 40 nm, although a few examples of larger agglomerates were found. The Pt/Al and Pt/Ce-Al catalysts seemed to contain also relatively small particles, but that did not result in better performance. The good performance of Au/Al and Pt-Au/Al catalysts could be at least partly related to the particle size distribution.

Based on the literature, both high acidity [11,12,25,45,46,47,48,49] and reducibility [12,25,49] contribute to enhanced activity in DCM oxidation. The reducibility of the catalysts had a substantial effect on the performance also in this study but in the opposite way. The catalysts with the lowest H_2_ consumption, i.e., Pt-Au/Al and Au/Al (see Table 2), demonstrated the best performance, whereas a high reducibility correlated with a poorer performance. Overall, the catalysts that demonstrated reducibility below 300 °C due to the presence of ceria (Pt/Ce-Al and Pt-Au/Ce-Al), except the Au/Ce-Al catalyst, likely because of the large Au particles that are unable to dissociate hydrogen, showed poorer performance compared to the less reducible catalysts.

Three least-acidic catalysts were among the three most active catalysts in DCM oxidation. When a weak acidity was considered, the acidity order of the catalysts from the lowest to highest was Pt-Au/Ce-Al that had the lowest acidity overall < Au/Al followed by the Pt-Au/Al catalyst < Au/Ce-Al < Pt/Ce-Al < Pt/Al. The Au/Al catalyst also had the highest amount of strong acid sites, which correlates with the performance in respect to the previous literature. It is possible that, in our case, the deposition of Au and/or Pt on ceria-alumina supports compensates the acidity loss to some extent by the introduction of Cl^−^ species from the chloride precursors used in the catalyst preparation. In addition, these acid sites could have different characteristics (Brønsted/Lewis acid sites) in different proportions as a result of the deposition, thus influencing the catalyst performance. The total basicity of the catalysts varied between 85 to 160 μmol g^−1^, but a direct correlation with the performance could not be seen. However, it is worth mentioning that the addition of Au in the Pt catalysts increased the basicity, and the DCM conversion was always higher in comparison to the monometallic Pt catalysts.

By considering the catalytic performance of the studied catalysts on previous findings, Chen et al. reported T_50_ and T_90_ values of approximately 220 °C and 250 °C, respectively, for a 5%Au/Co_3_O_4_ catalyst in DCM oxidation (DCM 500 ppm, 0.6 wt.% H_2_O, GHSV 15,000 h^−1^) [24]. In a study by Matêjová et al., a 0.3-Au/CeZr catalyst showed T_50_ and T_90_ values of 417 °C and 487 °C, respectively, in DCM oxidation (DCM 1000 ppm, 1.5 vol.% H_2_O, space velocity (SV) 71 m^3^ kg^−1^ h^−1^), with a maximum HCl yield of 77% [25]. Redina et al. showed that an Au-Rh/TiO_2_ catalyst reached a DCM conversion of 99% and HCl selectivity of 90% at 400 °C in DCM oxidation (DCM 510 ppm, H_2_O 0.25 vol.%, 40,000 h^−1^) [26].

### 2.3. Selectivity in DCM Oxidation

**Figure 6:**
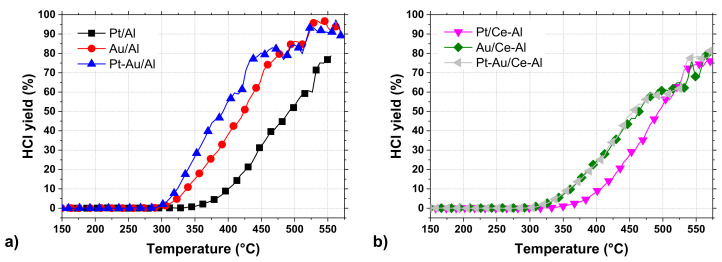
The HCl yields of the Pt, Au, and Pt-Au catalysts supported on (**a**) Al_2_O_3_ and on (**b**) CeO_2_-Al_2_O_3_ in DCM oxidation (same conditions as in Figure 5).

**Figure 7:**
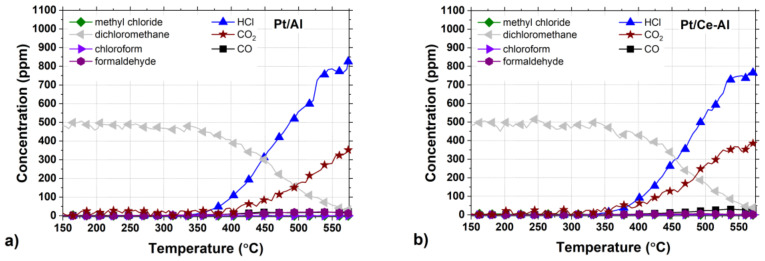

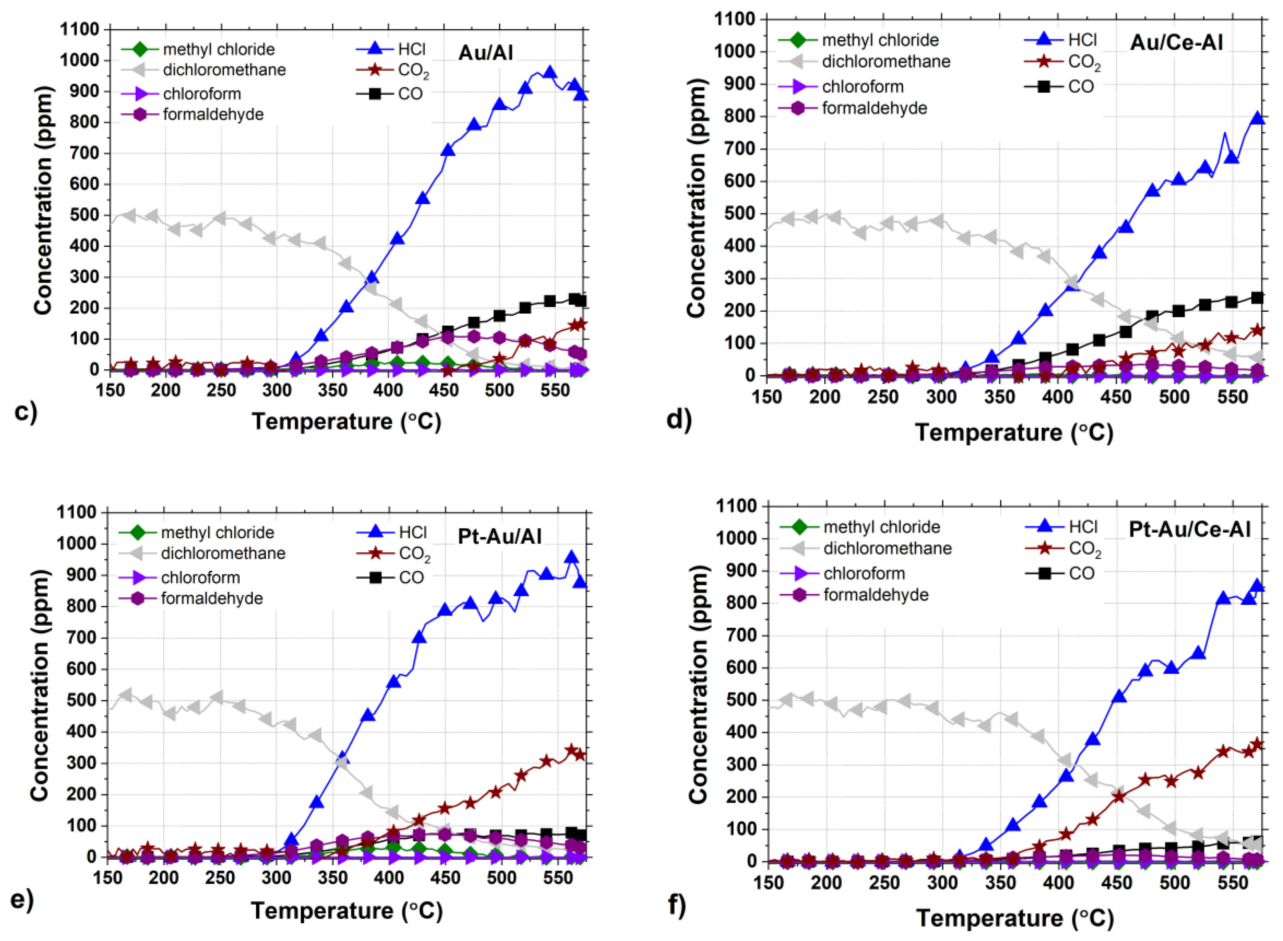
The product concentrations of the Pt, Au, and Pt-Au catalysts supported on (**a**–**c**) Al_2_O_3_ and on (**d**–**f**) CeO_2_-Al_2_O_3_ in DCM oxidation (same conditions as in Figure 5).

In addition to good catalytic performance, high selectivity is also essential in CVOC oxidation in order to avoid the formation of highly toxic by-products. An analysis of the reaction products confirmed that the main reaction products detected during DCM oxidation in this study were CO_2_, CO, and HCl. The formation of methyl chloride (CH_3_Cl) and formaldehyde (CH_2_O) were detected during the tests over certain catalysts at temperatures above 300 °C. The HCl yields of the studied catalysts are shown in Figure 6a,b and Table 4. The by-product formation for each catalyst is presented in Figure 7a–f.

The by-product formation for each catalyst is presented in Figure 7a–f. Amongst all the tested catalysts, Pt-Au/Al and Au/Al catalysts were the most active and HCl selective, both reaching close to 100% HCl yields at around 550 °C. At the same time, the other catalysts were able to reach HCl yields up to 70–80% during the tests. Even though the monometallic Au/Al and Au/Ce-Al catalysts were noticeably more active than the Pt catalysts, the formation of CO and other intermediates were observed to be substantial (Figure 7c,d). The Pt-Au/Al catalyst yielded mostly CO_2_ and HCl, but a slight formation of CO and formaldehyde was observed. No Cl-containing intermediates were observed during the experiment with the Pt-Au/Ce-Al catalyst. The catalysts in this study were synthesized from chloride-containing precursors and, therefore, may contain residual chlorine as prepared. Calcination in dry air is ineffective to eliminate residual chlorine as HCl. [50,51] Chlorine stays on alumina-based catalysts and, further, has an effect on hydrocarbon oxidation as an inhibitor. Residual chlorine is removed during oxidation when water is present, forming HCl, restores the catalytic activity, and may even increase the activity, because the initial Cl in contact with the active phase is eliminated completely [50].

The formation of carbon monoxide (CO) was observed in every case between concentrations of a few ppm up to over 200 ppm, depending on the catalyst. The Pt/Al and Pt/Ce-Al catalysts showed always the lowest CO formation, the maximum being 27 ppm at 555 °C and 30 ppm 530 °C, respectively. Over the Pt-Au/Al and Pt-Au/Ce-Al catalysts, the highest measured CO concentrations were 78 ppm at 560 °C and 62 ppm at 570 °C, respectively. The highest concentrations of CO amongst the studied catalysts were measured with the Au/Al and Au/Ce-Al catalysts, being over 200 ppm at temperatures higher than 520 °C. The CO formation started always at the same temperature levels as the HCl formation.

The Pt catalysts were the most selective towards the total oxidation product, i.e., carbon dioxide (CO_2_) (Figure 7a,b). The reason might be simply the well-known efficiency of Pt in complete oxidation and relatively low tendency to catalyze partial oxidation [52,53]. The bimetallic Pt-Au/Al and Pt-Au/Ce-Al catalysts were able to oxidize carbon intermediates better in comparison to monometallic Au catalysts (Figure 7e,f), which is probably due to the presence of Pt.

The selectivities towards HCl were rather high with all the catalysts, as evidenced by the methyl chloride concentrations, i.e., intermediate of the DCM oxidation reaction, that were relatively low, varying from zero up to 31 ppm. The highest methyl chloride concentrations were detected with the Pt-Au/Al catalyst, the maximum being 31 ppm at 405 °C followed by the Au/Al catalyst producing 27 ppm at the highest at 390 °C. The methyl chloride formation was not detected with the Pt-Au/Ce-Al catalyst at all, and with the other Ce-Al-supported catalysts and the Pt/Al catalyst, the concentrations were below 8 ppm. Methyl chloride formation was reported over alumina-supported catalysts in oxidative conditions earlier in several studies [10,11,17,18,19,45,46]. The presence of ceria seems to decrease the formation of methyl chloride.

Formaldehyde (CH_2_O) formation was observed with all the catalysts, and the maximum concentrations were from 6 ppm up to 108 ppm. The highest formaldehyde concentrations were observed over the Au/Al catalyst (>100 ppm between 440–505 °C) and the Pt-Au/Al catalyst (74 ppm at 430 °C). The lowest concentrations were seen over the Pt/Ce-Al (6 ppm at 495 °C), Pt-Au/Ce-Al (21 ppm at 445 °C), Au/Ce-Al (35 ppm at 470 °C), and Pt/Al (36 pp at 510 °C) catalysts. In this case, also, the presence of ceria decreased the formation of the intermediate.

Alumina support alone converted DCM selectively into HCl, reaching yields higher than 90% at temperatures above 555 °C (Table 4). However, methyl chloride (CH_3_Cl) formation up to 60 ppm was observed during oxidation, and carbon was detected as partial oxidation products (CO and formaldehyde) up to 530 °C, after which, CO_2_ formation started. The ceria-alumina support alone was also selective to HCl; the detected concentrations of other Cl-containing products were less than 7 ppm during the light-off test. In addition, the final carbon products were mainly CO_2_ and CO. In this case, also, the beneficial effect of ceria was visible. The formaldehyde formation was negligible. However, both supports demonstrated low DCM conversion. The alumina support did not reach T_90_, whereas with the ceria-alumina support T_90_ was 585 °C, as shown in Table 4.

The formation of methyl chloride in oxidative conditions over Al_2_O_3_-supported catalysts has been reported previously by several authors [11,12,18,19,45]. It has also been suggested that methyl chloride is formed in the presence of Lewis acid sites that are typical for Al_2_O_3_. Formaldehyde is reported to be formed on the Brønsted acid sites, which amounts could be increased in the presence of water [11,48]. The largest differences were seen in the case of CO formation, since both the Au/Al and Au/Ce-Al catalysts produced relatively high amounts of CO, i.e., over 200 ppm, in addition to the alumina support that produced CO over 340 ppm. The Pt-containing catalysts demonstrated the most beneficial product distributions in terms of the intermediate yields. The monometallic Au catalysts showed the lowest CO_2_ yields.

Oxygen activation is known to be easier on ceria and noble metals compared to alumina [54], which enables a faster delivery of reactive oxygen and, thus, accelerates total oxidation. In addition, surface diffusion and the strength of chemisorption on the catalyst influence the ability of the surface intermediates to move closer to each other for further reactions [12]. The oxygen activation (^18^O_2_-TPIE), which was discussed in detail for monometallic catalysts in Reference [27] and for bimetallic catalysts in Reference [28], correlates well with the DCM oxidation in the case of the Au/Al and Pt/Al catalysts. Oxygen exchange starts at around 310 °C with the Au/Al catalyst, i.e., at the same temperature as the formation of HCl and partial oxidation products (7 ppm of HCl and 10 ppm of CH_2_O at 300 °C). A low formation of ^18^O^16^O (0–0.1 mbar) was seen already from the beginning of the experiment between 200–310 °C, after which, it rapidly increased. The Pt/Al catalyst activated oxygen based on the formation of ^18^O^16^O at around 370 °C (first observations of ^18^O^16^O, i.e., 0–0.1 mbar, already at around 340 °C) and during DCM oxidation; 6 ppm of HCl and 5 ppm of CH_2_O were observed at 340 °C. Although the oxygen activation with the Pt-Au/Al catalyst started at a higher temperature of roughly 380 °C, oxidation products during DCM oxidation were observed already at around 290 °C (4 ppm of HCl and 11 ppm of CH_2_O), i.e., at the same temperature where the low formation of ^16^O^16^O (0–0.1 mbar) was seen in the ^18^O_2_-TPIE experiment. The Au/Ce-Al catalyst started oxygen activation progressively at 380 °C, but a low formation of ^18^O^16^O (0–0.1 mbar) was observed already between 250 to 380 °C. During DCM oxidation, the formation of HCl and oxidation products were seen at 305 °C (5 ppm of HCl and 6 ppm of CH_2_O). In the case of the Pt/Ce-Al catalyst, the oxygen activation began gradually at 340 °C, and a low formation of ^18^O^16^O was observed at above 250 °C, approximately. During DCM oxidation, the formation of HCl and CO_2_ was seen at the same temperature (345 °C). The oxygen activation seems to have a connection to the initiation of the DCM oxidation reaction with alumina-supported catalysts. With the Pt-Au/Ce-Al catalyst, the oxygen activation started at around 390 °C, but during DCM oxidation, the formation of HCl, CH_2_O, and CO_2_ was seen already at 315 °C, which cannot be explained by the ^18^O_2_-TPIE experiment. This could be related to the reduction of Pt-Au/Ce-Al, which demonstrated an H_2_ uptake between 150–300 °C. The behavior of the ceria-alumina catalyst could be connected to the reducibility of ceria, i.e., ability to gain electrons that provide the available lattice oxygen already at lower temperatures before the oxygen activation starts to increase progressively. Au was in the metallic state in all the Au containing catalysts, except in the Au/Al catalyst, and no significant differences were evidenced based on the XPS results. Thus, the oxidation state of Au does not seem to explain the differences in the catalytic performance of Au/Al and Pt-Au/Al catalysts in comparison to the Au/Ce-Al and Pt-Au/Ce-Al catalysts.

**Figure 8:**
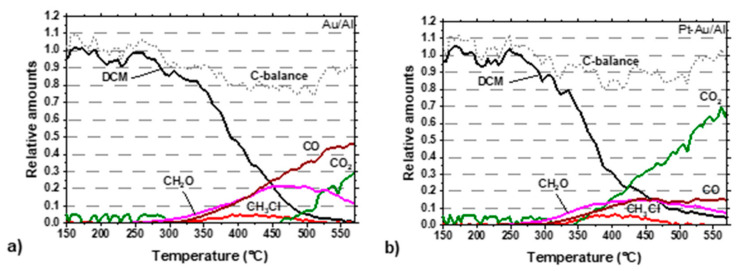
DCM oxidation over (**a**) Au/Al and (**b**) Pt-Au/Al catalysts (the same conditions as in Figure 5).

Carbon balances for the best performing catalysts during the light-off experiments are shown in Figure 8a,b. In the case of the Au/Al catalyst, the CO_2_ formation started at 450 °C, which did not directly have an effect on the CO formation, but formaldehyde formation began simultaneously to decline. The carbon balance nearly enclosed at the end of the experiment. The Pt-Au/Al catalyst showed clearly a better performance towards total oxidation, as seen in Figure 8b, compared to the Au/Al catalyst in Figure 8a. CO_2_ formation started at around 340 °C, increasing towards the higher temperature area at the end of the experiment. In addition, CO and formaldehyde formation was seen during the whole temperature range of the reaction, but both yields were below 16%. Carbon balance enclosed at the end of the experiment.

### 2.4. Stability of Pt-Au/Al Catalyst

The durability of a catalyst is of great importance, especially in oxidation of chlorinated compounds. The most active and selective catalyst in this study, i.e., Pt-Au/Al, was chosen for a 100-h stability test. The experimental conditions were selected based on the T_90_ temperature, but in isothermal conditions, 90% DCM conversion was reached at a lower temperature. The conditions were as follows: DCM 500 ppm and a constant temperature of 395 °C in moist conditions (1.5 vol.% water). During every refill of the DCM and water syringes, the temperature increased roughly by 10 °C, because no gas was fed into the reactor (by-pass) but stabilized down to 395 °C in a few minutes after the gas stream was fed back into the reactor.

**Figure 9:**
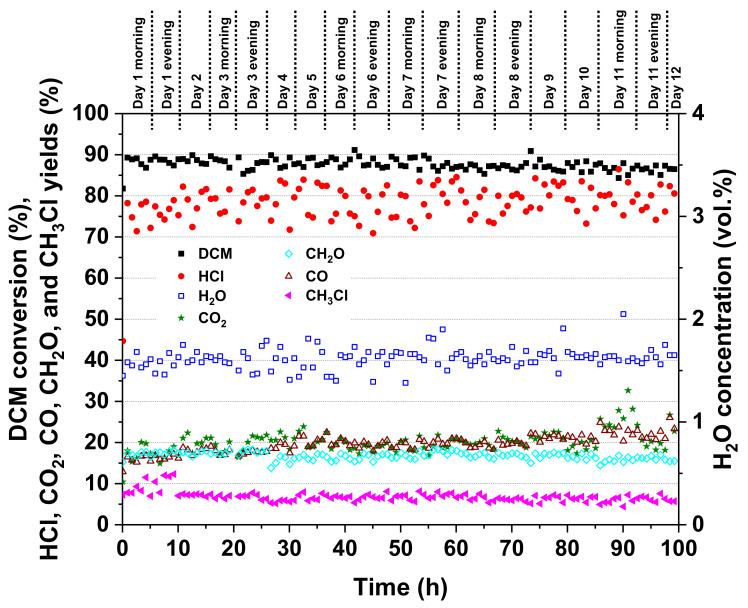
Stability of the Pt-Au/Al catalyst in DCM oxidation during the 100-h test (395 °C, DCM 500 ppm, H_2_O 1.5 vol.%, weight hourly space velocity (WHSV) 720 g g_cat_^−1^ h^−1^).

Based on the results shown in Figure 9, the performance of Pt-Au/Al was stable throughout the 100-h test. The DCM conversion remained between roughly 85% and 93%. Only a slight decline in the DCM conversion was observed after 50 h. At the same time, the HCl yield seemed to increase by a few percent units on average. The HCl yield fluctuated from 68% to 91% and the CO_2_ yield from 13% to 33%. Considering by-products, the CO and formaldehyde yields were from 14% to 30% and 12% to 20%, respectively. Methyl chloride (CH_3_Cl) formation seemed to decline by a few percent.

**Figure 10:**
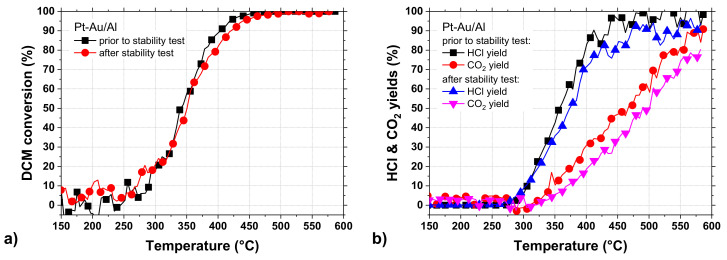
(**a**) DCM conversion, as well as (**b**) HCl and CO_2_ yields, over the Pt-Au/Al catalyst before and after the stability test in DCM oxidation (same conditions as in Figure 5).

The light-off experiments performed before and after the stability test (Figure 10a,b) indicated a slight sintering of active sites and/or support material based on the shape of the curves in Figure 10a [55]. The T_50_ and T_90_ values did not change substantially as the values increased by 5 °C and 20 °C after the stability test, respectively. The high-resolution transmission electron microscope (HR-TEM) and high-angle annular dark-field scanning transmission electron microscope (HAADF-STEM) analyses done after the 100-h stability test provided evidence for the sintering of bimetallic Pt-Au particles. Based on Figure 9, the CO_2_ and CO formation showed a minor increase during the 100-h stability test, but the light-off experiment performed after the stability test (Figure 10b) showed a lower CO_2_ formation. This might be due to the differences between experiments conducted at constant and increasing temperatures.

**Figure 11:**
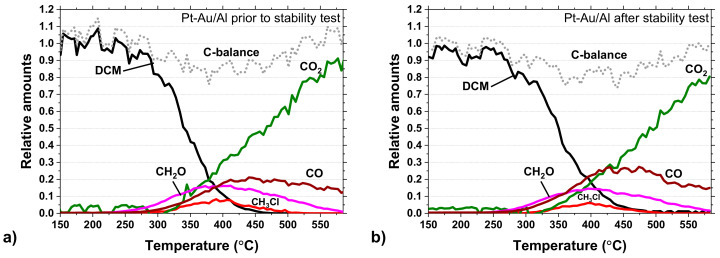
Carbon balance in DCM oxidation over the Pt-Au/Al catalyst (**a**) prior to and (**b**) after the stability test (same conditions as in Figure 5).

Carbon balances for the Pt-Au/Al catalyst during the light-off tests before and after the 100-h stability test are shown in Figure 11a,b. Signs of deactivation can be seen based on the yields of CO_2_ and CO. The CO_2_ yield is slightly lower and, in turn, the CO yield higher in Figure 11b. In this regard, the stability test might cause changes in the catalyst structure, affecting the oxidation ability of the Pt-Au/Al catalyst. Yet, the methyl chloride (CH_3_Cl) formation was a bit lower, and this was also seen during the stability test (Figure 9), as mentioned earlier. The carbon balance enclosed in both light-off experiments.

Chen et al. [24] studied the stability of a catalyst loaded with 5% of Au on Co_3_O_4_ in DCM oxidation (DCM 100 ppm, H_2_O 0.6 wt.%, balance air, GHSV 90,000 h^−1^, 350 °C). During 130 h on stream, the Au/Co_3_O_4_ catalyst did not show serious signs of deactivation at a DCM conversion level of around 90% to 95%, although a small decline could be observed similarly to our experiment. It is good to note that, in their study, the DCM concentration was five times lower and the Au loading five times higher compared to our experiments. Aida et al. [22] investigated the stability of Au/Al_2_O_3_ (5 wt.% of Au) in methyl chloride oxidation (catalyst 1 g, CH_3_Cl 1000 ppm, H_2_O 2 vol.%, balance air, weight/flow (W/F) 13.4 kg s mol^−1^, 300 °C). At a constant temperature of 300 °C, the catalyst lost its activity completely from around 100% conversion to 5% in 5 h, but it could be regenerated when raising the temperature above 427 °C. Based on thermogravimetric analysis done after the reaction, HCl did not desorb from the surface at temperatures below 327 °C, even after the addition of water, and the active sites suffered from deactivation. Once the temperature was increased above 400 °C, the catalyst activity recovered. The reaction of Cl and water, followed by the formation and further desorption of HCl, closed the catalytic cycle [22]. It is known that, in the catalytic oxidation of CVOC, it is essential to prevent the strong interaction of HCl with the catalyst surface, leading to deactivation [53], but at higher temperatures, another problem arises, since HCl attacks the alumina support [24]. Bond and Rosa [10] suggested that the reaction mechanism involves the disruption of the chlorinated reactant at a Lewis acid site, followed by the formation of metal-chloride bonds. After that, the chemisorption of water at a similar Lewis acid site and its reaction with the metal-chloride bond to form HCl takes place. Water and the reactant compete for the same sites, the water molecule more effectively. If the rate of formation of the Cl-M bonds is too high and the water vapor pressure too low, species such as Cl_2_M and Cl_3_M are formed, and these may react further to give gaseous metal chloride [10].

Legawiec-Jarzyna et al. [56] studied the catalytic activity of Pt-Au/Al_2_O_3_ catalysts in the hydrodechlorination of CCl_4_ and suggested that the introduction of Au onto Pt without a noticeable change in metal particle size decreases the affinity to the chloride species. A direct comparison of free energy of the formation for AuCl_3_ and PtCl_3_ (at the temperature range of 27–227 °C) evidence a much lower metal-Cl bond strength for gold. A higher activation energy for the monometallic (56 vs. ~30 kJ/mol for the bimetallics) indicates that the Cl removal from Pt surface is more difficult than for the bimetallic Pt-Au surface. Pt particles are very prone to excessive chloriding and, in effect, to a drastic deactivation in the hydrodechlorination of CCl_4_ [56].

**Figure 12:**
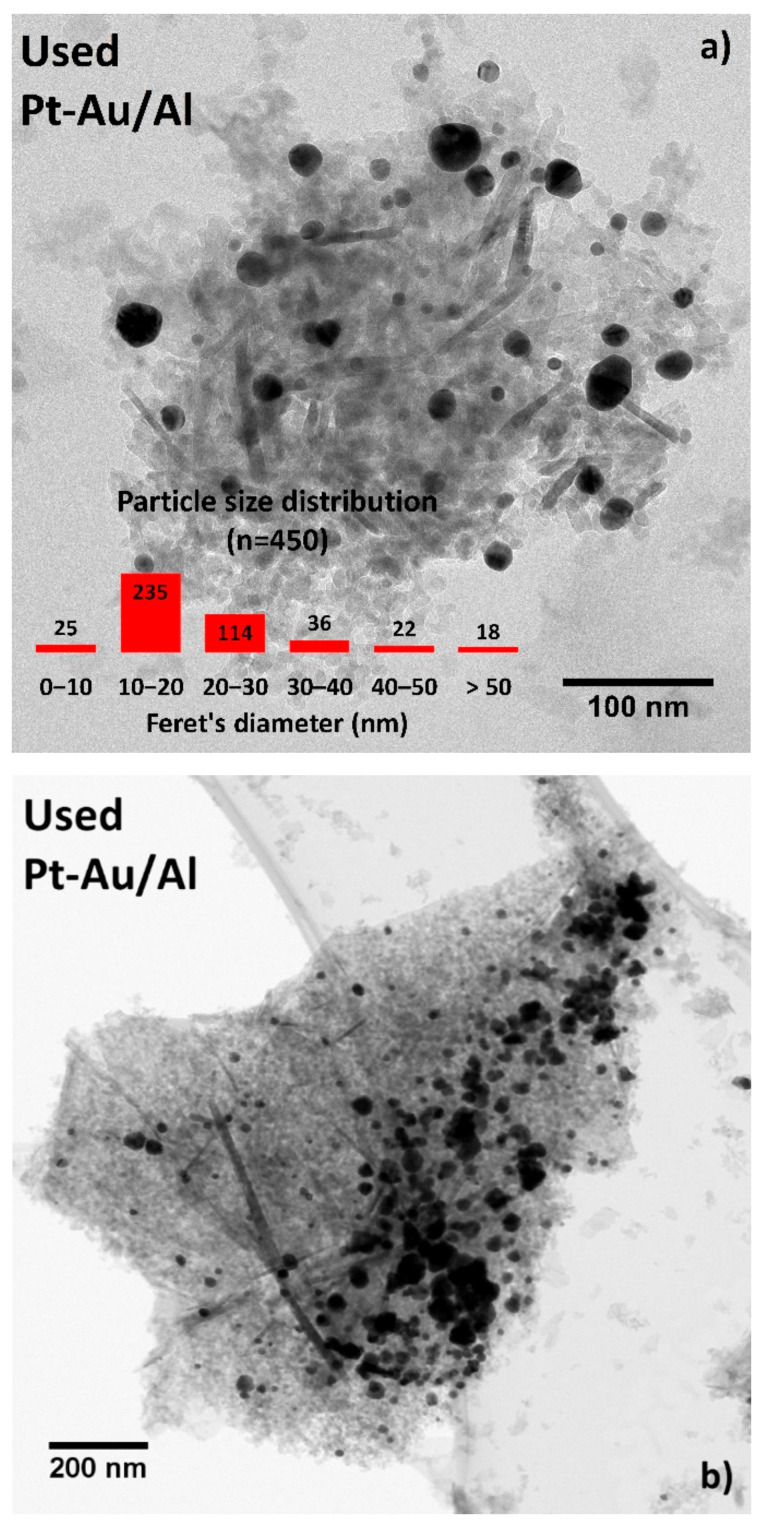
Representative (**a**) HR-TEM image with PSD data and (**b**) STEM image of the used Pt-Au/Al catalyst after the 100-h stability test in DCM oxidation (same conditions as in Figure 9).

Figure 12a shows a characteristic HR-TEM image with the corresponding PSD data that gave information on the structural changes in the catalyst after long-term testing. The bright-field STEM image shown in Figure 12b evidences the sintering of the active phases. The Feret’s diameter of the biggest agglomerates in that image were 150 to 175 nm. Even though the image provided proof of substantial sintering, the PSD data (*n* = 450) indicated the contrary. The amounts of particles in sizes between 0 to 10 nm and 10 to 20 nm increased by 2% and 9%, respectively, whereas the amounts in sizes between 20–30 nm decreased by 20% after the stability test. Although, the amounts of larger particles (i.e., between 30 to 40 nm, 40 to 50 nm, and >50 nm) increased all by a few percent units, the distribution indicated a larger number of smaller particles, mainly between 10–20 nm (52%) in comparison to the fresh Pt-Au/Al catalyst with 43% for the same size range. These findings might be a result of the removal of the residual chlorine. There might not be enough chlorine present on the catalyst surface to induce severe sintering of the particles. Au having resistance against highly electronegative elements and the reduced amount of Cl in the catalyst during the reaction might be one reason to maintain the catalytic performance in the stability test.

### 2.5. The Role of Au in the Reaction Mechanism on Alumina-Supported Catalyst

The alumina-supported catalysts were chosen for further consideration regarding the reaction mechanism, since these were better than ceria-containing analogs. The main reaction products observed during DCM oxidation in this study were CO_2_, CO, and HCl (see Figure 7). The formation of methyl chloride (CH_3_Cl) and formaldehyde (CH_2_O) were detected during the tests over the Au/Al and Pt-Au/Al catalysts. In the case of Pt/Al, only formaldehyde was observed. To understand better the role of gold in DCM oxidation, measured gaseous products for the Pt/Al, Au/Al, and Au-Pt/Al catalysts are shown in Table 5. With the Pt/Al, Au/Al, and Pt-Au/Al catalysts, the formation of formaldehyde was seen already at about 40 °C lower temperature compared to HCl. This could mean that, at lower temperatures, direct DCM hydrolysis into formaldehyde takes place. Furthermore, methyl chloride was not observed in the case of Pt/Al, which could mean that the direct hydrolysis reaction rate is high, with Pt/Al also at a higher temperature. The DCM oxidation over the Pt/Al, Au/Al, and Pt-Au/Al catalysts in excess hydrogen originating from the water feed can advance via the cleavage of all chlorines from the carbon atom, thus following the lowest bond energy. Formaldehyde formation is expected to occur on Brønsted acid sites [48]. According to van den Brink et al. [11], the first step in DCM oxidation over γ-Al_2_O_3_ is the reaction of the adsorbed DCM molecule with a surface OH group that produces a chloromethoxyl species, which further reacts to the chemisorbed formaldehyde analog. The adsorbed formate species decomposes to CO or CO_2_. The chloride displaced after the formation of the chemisorbed formaldehyde species can react with surface Al^3+^ to form an aluminum chloride species or attach to a proton to form HCl. Water is able to regenerate the Al-Cl entities. [11].

Haber et al. and van den Brink et al. focused on the role of Lewis acid sites in DCM oxidation. Haber et al. suggested that the activity of alumina in DCM oxidation is related to the distribution of Lewis acid sites, whereas the formation and selectivity to methyl chloride is dependent on the concentration of Lewis acid sites [45,46]. In the mechanism proposed by van den Brink et al. [11], both chlorines of DCM are cleaved, and instead of forming HCl or aluminum chloride, Cl can also re-enter a surface methoxy species by nucleophilic displacement. The formaldehyde species, which was formed at a lower temperature range, can also disproportionate to form methoxy and formate groups. The methoxy species can react with HCl to form methyl chloride, which was observed in our case, at a slightly higher temperature. At higher temperatures, formaldehyde species can decompose rapidly and desorb more easily, making disproportionation less likely, and if methyl chloride is still formed, it will be decomposed on γ-Al_2_O_3_ [11].

Maupin et al. [19] suggested a slightly different mechanism for methyl chloride formation compared to that of van den Brink et al. [11]. Firstly, one chlorine atom from DCM is substituted by an alumina hydroxyl group (step 1), leading to the formation of chloromethoxy species. Secondly, the last species is transformed into a hemiacetal species (step 2). [19] These steps are in accordance with van den Brink et al. [11], with the exception that, in the mechanism by van den Brink et al., both chlorines of DCM are removed and then Cl re-enters on a surface methoxy species. However, the methyl chloride formation is explained differently. Maupin et al. [19] suggested that two chloromethoxy species react, via hemiacetal species, to form a formate species and methyl chloride (step 3), as discussed also by van den Brink et al., in which the hydride is transferred from the hemiacetal species rather than from the surface, as suggested by Haber et al. [45]. The chloromethoxy disproportionation is accompanied by the formation of Al-O-Al bridges that cause a decrease in the number of available hydroxyl groups. Then, the formed formate species decompose to CO or CO_2_, and finally, water regenerates the hydroxyl groups (step 4). [19]

Pinard et al. studied DCM oxidation over pure NaY and PtNaY catalysts [57], NaX and NaY zeolites [58], and PtHFAU [48] and PtFAU catalysts [59]. They reported that DCM transformation occurs bifunctionally via DCM hydrolysis on the Brønsted acid sites of the support, resulting in formaldehyde and HCl, followed by formaldehyde oxidation into CO_2_ and H_2_O on the Pt sites. Water is required for the formation of formaldehyde. Formation of CO may result from formaldehyde oxidation or by decomposition of formate species. The main reactions were suggested as:

CH_2_Cl_2_ + H_2_O→CH_2_O + 2HCl and (1)

CH_2_O + ½O_2_→CO + H_2_O (2)

A methyl chloride intermediate was formed at higher temperatures via the Lewis acid sites [48]. The CO formation could result from the direct oxidation of formaldehyde [48], in addition to the proposed decomposition of formate species on Lewis acid sites, as discussed by van den Brink et al. [11] and Maupin et al. [19]. According to Maupin et al. [19], Pt/Al_2_O_3_ catalysts oxidize DCM completely at 380 °C. The DCM reaction rate limiting step occurs on alumina, since neither the Pt content nor the dispersion (particle size) influence the conversion rates and selectivity [19]. Similar observations were seen in our previous study [12]. The addition of Pt and/or ceria to the catalyst improved the selectivity towards CO_2_, but the oxidation rates were not affected that much [12]. In our experiments, the addition of Au on alumina resulted in a significant decrease in the light-off temperature, and the addition Au on Pt/Al_2_O_3_ enhanced both DCM conversion and selectivity towards the total oxidation products.

Au catalysts are known to be effective in selective partial oxidation [42,43,60,61,62], often succeeding where other metals fail due to a weaker adsorption of the reactants (such as hydrocarbons) on the catalyst surface [63]. The too-weak adsorption of reaction intermediates might be one reason for the lower total oxidation result observed in our experiments with the monometallic Au catalysts. According to Haruta et al. [42], the surface adsorption and reactivity of Au can be modified by generating surface structures via downsizing or scratching. Due to the moderate bonding strength on the defect sites of Au, which is weaker than that on Pd and Pt, it often occurs that Au catalysts are better than other noble metal catalysts at low temperatures [42]. This can also be seen in our case, since reaction products are observed at lower temperatures with the Au-containing catalysts than with the Pt/Al.

In the presence of water, alumina is strongly hydrated, which facilitates the reaction between DCM and hydroxyl groups from alumina [19]. It is worth noting that alumina is also hydrophilic and, thus, attracts water on its surface [64]. In addition, the Pt/Al_2_O_3_ catalyst is hydrophilic [53], and Au has a hydrophilic nature [42]. Water is weakly adsorbed on Au [65]. The adsorption of water is followed by water dissociation into protons and OH groups, which are the sites for DCM adsorption, and, thus, the adsorption is enhanced. We could speculate that the relatively high amounts of formaldehyde produced by the Au/Al catalyst might result from a high amount of Brønsted acid sites. Costello et al. [66,67] studied an Au/γ-Al_2_O_3_ catalyst in CO oxidation and proposed that water forms Au^+^-OH^-^ via adsorption on Au clusters. Chemisorbed oxygen on Au has a Brønsted base character [42,65]. On the Au surface, atomic oxygen reacts like a Brønsted base by abstracting a hydrogen atom from acidic molecules. Furthermore, oxygen atoms on the Au(110) surface were observed to react like a nucleophilic base towards formaldehyde. [65] This might explain the product distributions in the case of the Au-containing alumina catalysts. Further oxidation of an adsorbed compound (in this case, formaldehyde) is suppressed when the activation energy for desorption is lower than that for oxidation. Ceria-containing catalysts, however, are able to oxidize carbon products, further resulting in the formation of CO and CO_2_ due to its high oxygen storage capacity and good redox property [68]. The different product distribution might be also a consequence of different reaction routes: on alumina, direct DCM hydrolysis into formaldehyde appears, and, on ceria, the Mars-van Krevelen mechanism prevails.

The role of Au is evident, since formaldehyde, methyl chloride, and CO are formed in comparison to Pt/Al, which produces mainly CO_2_. Au enhances the DCM conversion and HCl yield but results in partial oxidation products. The results suggest that DCM decomposition over the Au-containing alumina-supported catalysts proceeds via a bifunctional reaction in which hydrolysis is the first step, followed by oxidation.

## 3. Materials and Methods

### 3.1. Catalysts

Supported monometallic Pt and Au catalysts were prepared using wet impregnation and deposition-precipitation with urea, respectively. Bimetallic catalysts were prepared with surface redox reactions in an aqueous phase, and calcined monometallic Pt catalysts were used as “parent” catalysts in their synthesis. Commercial aluminum oxide (γ-Al_2_O_3_, Rhodia) was used as the support material and as a basis for the preparation of the ceria-alumina (20 wt.% of ceria) support (wet impregnation). Nomenclature and target compositions of the catalysts are shown in Table 6. Calcination of the catalysts was done in air for 5 h at 600 °C with a heating rate of 5 °C min^−1^. The preparation steps are described in detail in the Supplementary Materials.

### 3.2. Catalyst Characterization

The prepared catalysts were characterized using ICP-OES (Perkin Elmer Optima 2000 DV, Waltham, MA, USA), N_2_ physisorption (Micromeritics ASAP 2020, Norcross, GA, USA), X-ray diffraction (XRD; Siemens D5000, Munich, Germany), X-ray photoelectron spectroscopy (XPS; Thermo Fisher Scientific ESCALab 250Xi, Waltham, MA, USA), high-resolution transmission electron microscope (HR-TEM), temperature-programmed reduction with hydrogen (H_2_-TPR), temperature-programmed isotopic exchange (TPIE) of labeled oxygen (^18^O_2_), and temperature-programmed desorption of ammonia (NH_3_-TPD) and carbon dioxide (CO_2_-TPD). Details of the characterization procedures and the results are given in References [27,28].

In order to study the properties of the catalysts further, high-resolution transmission electron microscope (HR-TEM) and high-angle annular dark-field scanning transmission electron microscope (HAADF-STEM) were employed. HR-TEM and HAADF-STEM images were obtained using two devices: a JEOL JEM-2100 (Tokyo, Japan) and a JEOL JEM-2200FS (Tokyo, Japan), both operated at an acceleration voltage of 200 kV and equipped with an energy-dispersive X-ray (EDX) spectrometer (JEOL, Tokyo, Japan). HR-TEM and HAADF-STEM (JEOL JEM-2200FS) were used as the characterization methods after a 100-h stability test in order to examine the surface of the Pt-Au/Al catalyst. Temperature-programmed desorption (NH_3_-TPD and CO_2_-TPD) experiments were done for the monometallic Au and Pt catalysts supported on alumina and ceria-alumina. The NH_3_-TPD experiments were done using a Quantachrome Chembet Pulsar TPR/TPD device (Boynton Beach, FL, USA) equipped with a thermal conductivity detector (TCD). The samples (200 mg) were pretreated under an He flow at 500 °C for 30 min and were cooled down to 100 °C in an He flow. Then, the samples were treated with NH_3_ for 60 min at 100 °C using 120 mL min^−1^ of 10% NH_3_ in He. The physiosorbed NH_3_ was removed by feeding He for 30 min at 100 °C. Finally, NH_3_ desorption was conducted from 100 °C to 950 °C (ramp rate of 10 °C min^−1^). The CO_2_-TPD experiments were done using an Autochem II device (Micromeritics, Norcross, GA, USA) equipped with a TCD detector. The samples (50 mg) were flushed in an He flow at 450 °C for 30 min. Next, 5% CO_2_ in He (50 mL min^−1^) was adsorbed on the sample over 60 min at 50 °C. After flushing with He for 30 min, the TPD was conducted from 50 to 600 °C at a heating rate of 10 °C min^−1^. XPS measurements were done using a Thermo Fisher Scientific ESCALab 250Xi spectrometer (Waltham, MA, USA) with an Al Kα (1486.6 eV) radiation source and a pass energy of 20 eV. The data were analyzed with Thermo Scientific Avantage™ software (v. 5.9904), and the signals were fitted with the mixed Gaussian–Lorentzian function. Binding energies were referred to the C1s peak line at 284.8 eV, and the Smart function was used to reduce the background.

### 3.3. Catalytic Experiments

Catalytic experiments were performed in a quartz reactor system operated under atmospheric pressure. The materials used in the experimental set-up (quartz glass, Teflon tubings heated to 180 °C, and Teflon connectors) are corrosion-resistant due to the nature of reaction products. Liquid DCM and water were injected into an evaporator using gas tight syringes and mixed with air fed through a mass flow controller. The preheating oven, filled with glass spheres to ensure the mixing of gases, was set to 150 °C. More specific descriptions of the experimental set-up and the Fourier-transform infrared (FTIR) analyzer (Gasmet Technologies, Helsinki, Finland) used in the gas analysis can be found in References [12,69]. The accuracy of the FTIR analysis was 2 ppm.

Catalytic experiments were carried out in a temperature range from 100 °C to 600 °C, with a heating rate of 5 °C min^−1^ and a total gas flow of 1 L min^−1^ equivalent to a weight hourly space velocity (WHSV) of 720 g g_cat_^−1^ h^−1^. The DCM concentration was set to 500 ppm and water feed to 1.5 vol.% in all the experiments. A catalyst sample of 100 mg was placed in the vertically aligned tubular fixed-bed reactor between quartz wool plugs to hold it in place. Before each experiment, the catalysts were pretreated in an air flow from room temperature to 600 °C, with the heating rate of 10 °C min^−1^. The light-off experiments were repeated once to verify the results. The 100-h stability test was carried out for the Pt-Au/Al catalyst at 395 °C, corresponding to ~90% DCM conversion. The continuous feed of DCM (500 ppm) into the evaporator unit was limited to approximately 5.5 h before the syringe needed to be refilled. The refills for DCM and water were done simultaneously, and their concentrations were let to stabilize before the gas stream (air + DCM + H_2_O) was fed back into the reactor. At the end of each testing day, the feed stream was stopped, and the oven was switched off for the night. The following day, after the oven temperature reached the set point, the stabilized gas stream was introduced into the catalyst bed again.

A Gasmet DX-4000N FTIR analyzer (Gasmet Technologies, Helsinki, Finland), capable of detecting close to all gas phase compounds except diatomic homonuclear compounds such as chlorine, hydrogen, oxygen, and nitrogen, was used for gas analysis. The analyzer was calibrated to detect the following chlorinated compounds: CH_2_Cl_2_, C_2_Cl_4_, C_2_HCl_3_, CHCl_3_, COCl_2_, CH_3_Cl, and HCl. In addition, different VOCs (such as formaldehyde, CH_2_O) and CO_2_, CO, NO_x_, as well as water, were included in the calibrations. Altogether, 41 compounds were analyzed. The conversion of dichloromethane (CH_2_Cl_2_, DCM), and the yields of hydrochloric acid (HCl) and carbon dioxide (CO_2_), which are the desired final products, were calculated as follows:

(3) $$X_{DCM}\;=100\times \frac{c_{DCM}^{in}-c_{DCM}^{out}}{c_{DCM}^{in}}$$

(4) $$\Upsilon_{HCl}\;=100\times \frac{c_{HCl}^{out}}{2\times c_{DCM}^{in}}$$

(5) $$\Upsilon_{CO_{2}}\;=100\times \frac{c_{CO_{2}}^{out}}{c_{DCM}^{in}}$$

where X is the conversion (%), Υ is the yield (%), $c_{x}^{y}$ is the concentration of the compound x (ppm) (x denoting DCM, HCl, or CO_2_, and y representing the inlet or outlet concentration).

## 4. Conclusions

Six metal loaded catalysts containing Au, Pt, and Pt-Au supported on Al_2_O_3_ and CeO_2_-Al_2_O_3_ were studied in the DCM oxidation. The results showed that Au/Al_2_O_3_ is active and highly HCl selective, even outperforming Pt/Al_2_O_3_, which was found as the most active and HCl selective catalyst in our previous study. The reasons might be the small Au particle size (~5 nm), with narrow size distribution (±5 nm) and good metal dispersion. The oxygen activation ability seems to lead to the high DCM conversion of Au/Al and Pt-Au/Al catalysts in DCM oxidation. Au changes the product distribution. The formation of Cl-containing intermediates such as methyl chloride was fairly low with the Pt-Au/Al catalyst and zero with the Pt-Au/Ce-Al catalyst. Based on our experiments, the total acidity of the catalysts decreased after the introduction of ceria to alumina, thus decreasing DCM adsorption and, consequently, the catalytic performance. At the same time, the addition of ceria effectively improved the selectivity towards the total oxidation products, decreasing the formation of intermediates such as methyl chloride, carbon monoxide, and formaldehyde. The results suggest that, with the Au-containing Al_2_O_3_-supported catalysts, DCM decomposition occurs mainly via direct DCM hydrolysis into formaldehyde and HCl, followed by the oxidation of formaldehyde into CO and CO_2_. To get deeper insight on the reaction mechanism, in situ studies are required.

The bimetallic Pt-Au/Al_2_O_3_ catalyst showed good catalytic performance and stability based on the light-off and 100-h stability experiments at 395 °C, but selectivity towards the total oxidation products in the stability test was not satisfactory. By-product yields, i.e., CO, formaldehyde, and methyl chloride, between 5% to 30% were observed. The results are significant in two respects. Firstly, they demonstrated the possibility of Au being used effectively in CVOC oxidation (high DCM conversion and HCl selectivity), and, secondly, the Pt-Au/Al catalyst showed chlorine resistance during the stability test. The next step is to study whether the DCM oxidation over Au catalysts is size- and/or shape-dependent and to confirm the role of reducibility and acidity, especially with respect to Brønsted and Lewis acid sites of the Au catalyst.

## Figures and Tables

**Table 1:** Summary of the previously published results in References [27,28] and the new results presented in this study. DCM: dichloromethane, ICP-OES: inductively coupled plasma optical emission spectroscopy, BET-BJH: Brunauer-Emmett-Teller/Barrett-Joyner-Halenda method, XRD: X-ray diffraction, XPS: X-ray photoelectron spectroscopy, HR-TEM: high-resolution transmission electron microscope, H_2_-TPR: temperature-programmed reduction with hydrogen, ^18^O_2_-TPIE: temperature-programmed isotopic exchange (TPIE) of labeled oxygen, NH_3_-TPD: temperature-programmed desorption of ammonia, and CO_2_-TPD: temperature-programmed desorption of carbon dioxide.

Results	Monometallic Catalysts	Bimetallic Catalysts
ICP-OES	Reference [27]	Reference [28]
BET-BJH	This study	Reference [28]
XRD	Reference [27]	Reference [28]
H_2_-TPR	Reference [27]	Reference [28]
^18^O_2_-TPIE	Reference [27]	Reference [28]
XPS	This study	Reference [28]
HR-TEM	This study	Reference [28]
NH_3_-TPD	This study	Reference [28]
CO_2_-TPD	This study/Reference [28]	Reference [28]
Catalytic performance in DCM oxidation	This study	This study
Stability in DCM oxidation	This study	This study

**Table 2:** Summary of the metal loading, surface area, total pore volume, average pore diameter, reducibility, and basicity of the tested catalysts. The results were discussed in detail in References [27,28]. The results marked with * are the new results originating from this study.

Catalyst	Metal Loading (wt.%)		S_BET_^a^ (m^2^ g^−1^)	Total Pore Volume ^b^ (cm^3^ g^−1^)	Average Pore Diameter ^b^ (nm)	H_2_ Consumption (µmol g^−1^)	CO_2_-TPD ^c^ (µmol g^−1^)
	Au	Pt					
Al_2_O_3_	-	-	100	-	-	-	-
Pt/Al	-	1.1	105 *	0.51 *	18 *	149	100 *
Au/Al	0.7	-	95 *	0.48 *	18 *	52	135 *
Pt-Au/Al	0.8	0.9	95	0.49	19	44	100
CeO_2_-Al_2_O_3_	-	-	65	-	-	-	-
Pt/Ce-Al	-	1.2	85 *	0.38 *	16 *	326	85 *
Au/Ce-Al	0.5	-	75 *	0.35 *	17 *	83	160 *
Pt-Au/Ce-Al	0.9	1.1	85	0.41	18	315	105

^a^ Round values (± 5 m^2^ g^−1^), ^b^ cumulative pore volume between diameters of 1.7 nm and 300 nm, and ^c^ round values (± 5 µmol g^−1^). - = not reached; S_BET_—BET surface areas.

**Table 3:** Surface composition of the Au-containing catalysts.

Catalyst	XPS				
	Surface Composition (wt.%)				
	Au	Pt	Ce	Al	O
Au/Al	1.6	-	-	49.6	48.9
Au/Ce-Al	0.6	-	6.3	46.5	46.6
Pt-Au/Al	0.9	1.2	-	51.3	46.6
Pt-Au/Ce-Al	2.2	2.1	4.4	48.1	43.2

**Table 4:** Summary of the catalytic performance and selectivity of all the tested catalysts in DCM oxidation (DCM 500 ppm, H_2_O 1.5 vol.%, weight hourly space velocity (WHSV) 720 g gcat^−1^ h^−1^).

Catalyst	T_50_ (°C)	T_90_ (°C)	HCl Yield at T_90_ (%)	Max. HCl Yield (%)	CO_2_ Yield at T_90_ (%)	Max. CO_2_ Yield (%)
Al_2_O_3_	455	-	-	96 ^a^	-	5 ^b^
Pt/Al	460	555	79	79	70	70
Au/Al	390	475	80	97	2	30
Pt-Au/Al	375	485	81	97	36	70
CeO_2_-Al_2_O_3_	470	585	76	76	48	48
Pt/Ce-Al	470	560	73	76	70	77
Au/Ce-Al	435	-	-	80 ^c^	-	28 ^d^
Pt-Au/Ce-Al	430	570	81	81	70	70

- = not reached; ^a^ DCM conversion at 570 °C = 89%, ^b^ CO_2_ yield at 570 °C, ^c^ DCM conversion at 570 °C = 89%, and ^d^ CO_2_ yield at 570 °C.

**Table 5:** The gaseous products detected during the light-off test (same conditions as in Figure 5).

	DCM Conv. at 570 °C (%)	CH_2_O (ppm at Temp. Range, °C)	CH_3_Cl (ppm at Temp. Range, °C)	CO (ppm at Temp. Range, °C)	CO_2_ (ppm at Temp. Range, °C)	HCl (ppm at Temp. Range, °C)
Pt/Al	90 at 555	2–35 at 285–555	-	2–30 at 340–555	2–350 at 290–555	2–795 at 330–555
Au/Al	100	2–110 at 250–575	2–30 at 300–575	2–230 at 305–575	2–150 at 460–575	2–960 at 290–575
Pt-Au/Al	96	2–75 at 245–570	2–30 at 315–495	2–80 at 265–570	2–340 at 345–570	2–955 at 290–570

**Table 6:** Abbreviations, desired metal loadings, and the used preparation methods for the studied catalysts.

Catalyst	Support	Desired Metal Loading (wt.%)		Preparation Method
		Au	Pt	
Pt/Al	Al_2_O_3_		1	Wet impregnation
Au/Al	Al_2_O_3_	1		Deposition precipitation with urea
Pt-Au/Al	Al_2_O_3_	1	1	Surface redox reactions in aqueous phase
Pt/Ce-Al	CeO_2_-Al_2_O_3_		1	Wet impregnation
Au/Ce-Al	CeO_2_-Al_2_O_3_	1		Deposition precipitation with urea
Pt-Au/Ce-Al	CeO_2_-Al_2_O_3_	1	1	Surface redox reactions in aqueous phase

## Supplementary Materials

The following are available online.
