# Supplementary material for: Oxidation of Dichloromethane over Au, Pt, and Pt-Au Containing Catalysts Supported on γ-Al2O3 and CeO2-Al2O3
Source: Molecules. 2020 Oct 12;25(20):4644. doi: 10.3390/molecules25204644 (PMC7587384; doi:10.3390/molecules25204644)
Supplement: Supplementary file 1 [file molecules-25-04644-s001.pdf]

Supplementary Material

Oxidation of dichloromethane over Au, Pt, and Pt-Au containing catalysts supported on γ-Al_2O_3 and CeO_2-Al_2O_3

Tuomas K. Nevanperä [1], Satu Pitkäaho [1], Satu Ojala [1], and Riitta L. Keiski [1,*]

[1] University of Oulu, Faculty of Technology, Environmental and Chemical Engineering, FI-90014 University of Oulu, P.O.Box 4300; firstname.lastname@oulu.fi

* Correspondence: riitta.keiski@oulu.fi; Tel.: +358 40 726 3018

Catalyst preparation

Two different support oxides, i.e., Al_2O_3 and CeO_2-Al_2O_3, were used in the preparation of six catalysts (Table 2). Commercial γ-Al_2O_3 (Rhodia) was used in the preparation of support containing 20 wt.% of CeO_2 by wet impregnation in excess solution according to Reference [1]. The metal salt used for the impregnation was cerium (III) chloride heptahydride ($CeCl_3 \cdot 7H_2O$, Acros Organics, 99%). A requisite amount of the metal salt was dissolved in distilled water (~25 mL) and mixed with 4 g of γ-Al_2O_3 in a rotary evaporator to obtain 5 g of the CeO_2-Al_2O_3 support. Next, the solution was stirred vigorously for 2 h at room temperature. Excess water was evaporated in the rotary evaporator at 60 °C. The obtained support material was dried further in an oven overnight at 120 °C [2].

The Pt/Al and Pt/Ce-Al catalysts were prepared using wet impregnation. Firstly, the γ-Al_2O_3 powder was wetted by distilled water in a beaker in order to have high dispersion by maximizing the mass transfer of added metal salt on the surface and pores of the catalyst. A requisite amount of metal salt ($H_2PtCl_6 \cdot 6H_2O$, Alfa Aesar, Pt ca 38-40%) was dissolved in ~20 mL of distilled water. The solutions were mixed together and stirred for 18 h at room temperature. Finally, the solution was dried on a sand bed at 60 °C and further in an oven at 120 °C overnight. A similar procedure was used in the preparation of the Pt/Ce-Al catalyst.

Deposition-precipitation with urea was used for loading 1 wt.% of Au on the support according to Reference [1]. Firstly, the support powder was dispersed in distilled water. The temperature of the suspension was kept at 80 °C, and the suspension was agitated with a magnetic stirrer. Secondly, a requisite amount of chloroauric acid ($HAuCl_4 \cdot 3H_2O$, Acros) solution was added into the suspension, and the temperature was let to stabilize. Thirdly, 4.5 g of urea (Aldrich, 99%) was added into the reactor vessel, and the suspension was stirred continuously for 4 h. The reactor vessel was covered thoroughly with Al foil to avoid UV light-induced effects. The deposition was followed by centrifugation of the catalyst suspension in 50-mL tubes. The centrifugation was done three times. After the first and second centrifugations, the separated water was decanted away, and the tube was refilled with distilled water. After the following separation and washing, the solid was collected and moved into a rotary evaporator and dried at ~40 °C in a water bath under vacuum. Final drying was done in an oven at 120 °C overnight. During handling and storage, the exposure of catalysts to light was minimized. The catalysts were stored at room temperature and kept away from light. The CeO_2-Al_2O_3 support and Au/Al, Au/Ce-Al, Pt/Al, and Pt/Ce-Al catalysts were calcined under a 10 vol.% O_2/N_2 flow by heating from room temperature up to 600 °C by increasing the temperature at a rate of 5 °C min^{-1} and dwelling at 600 °C for 5 h.

The calcined monometallic Pt catalysts were used as "parent" catalysts for the preparation of bimetallic Pt-Au catalysts. Bimetallic catalysts were prepared using surface redox reactions [3]. A requisite quantity of a monometallic parent catalyst was placed inside a glass reactor. The reactor was first flushed with a countercurrent N_2 flow for 15 min at room temperature. Next, the catalyst was reduced in an H_2 flow of 100 cm^3 min^{-1} by increasing the temperature at a rate of 5 °C min^{-1} up to 300 °C. After the reduction, the reactor was cooled down to room temperature, and ultrapure water was used to rinse the catalyst bed. Then, the reactor was degassed by feeding N_2 for 10 min at room temperature. A requisite amount of Au salt ($HAuCl_4 \cdot 3H_2O$, Acros) was dissolved in ultrapure water, and the pH of the solution was adjusted with HCl. The Au solution was fed into the reactor under an N_2 flow. The solution was kept in the reactor for 1 h under an H_2 flow of 300 cm^3 min^{-1}. The resulting bimetallic catalyst was separated from the aqueous solution with a

sieve. The samples were dried under a H_2 flow of 300 cm^3 min^{-1} at 100 °C overnight. Finally, the catalysts were reduced by increasing the temperature from 100 °C up to 300 °C for 1 h. The obtained bimetallic catalysts were calcined in a muffle furnace through heating from room temperature up to 600 °C at a heating rate of 5 °C min^{-1} and dwelling for 5 h under atmospheric conditions [4].

References

1. Lakshmanan, P.; Delannoy, L.; Richard, V.; Méthivier, C.; Potvin, C.; Louis, C. Total oxidation of propene over Au/$x$$CeO_2$-$Al_2O_3$ catalysts: Influence of the CeO_2 loading and the activation treatment. *Appl. Catal. B Environ.* **2010**, *96*, 117–125, doi:10.1016/j.apcatb.2010.02.009.
2. Nevanperä, T.K.; Ojala, S.; Bion, N.; Epron, F.; Keiski, R.L. Catalytic oxidation of dimethyl disulfide (CH_3SSCH_3) over monometallic Au, Pt and Cu catalysts supported on γ-Al_2O_3, CeO_2 and CeO_2-Al_2O_3. *Appl. Catal. B Environ.* **2016**, *182*, 611–625, doi:10.1016/j.apcatb.2015.10.012.
3. Epron, F.; Especel, C.; Lafaye, G.; Marécot, P. Multimetallic Nanoparticles Prepared by Redox Processes Applied in Catalysis. In *Nanoparticles and Catalysis*; Astruc, D., Ed.; Wiley-VCH Verlag GmbH & Co. KGaA: Weinheim, Germany, 2008; pp. 279–302.
4. Nevanperä, T.K.; Ojala, S.; Laitinen, T.; Pitkäaho, S.; Saukko, S.; Keiski, R.L. Catalytic Oxidation of Dimethyl Disulfide over Bimetallic Cu–Au and Pt–Au Catalysts Supported on γ-Al_2O_3, CeO_2, and CeO_2–Al_2O_3. *Catalysts* **2019**, *9*, 603, doi:10.3390/catal9070603.
